# Changes in the use of vitamin K antagonists and direct oral anticoagulants and impact on the incidence of oral anticoagulation-related intracerebral hemorrhage: population-wide prescription patterns in two 5-year cohorts

**DOI:** 10.1080/07853890.2026.2652115

**Published:** 2026-04-06

**Authors:** Liisa Tomppo, Hanne Sallinen, Otto Lankinen, Giannis Mastoras, Georgios Georgiopoulos, Sven Poli, Jukka Putaala, Daniel Strbian

**Affiliations:** a Department of Neurology and Neurosciences, Helsinki University Hospital and University of Helsinki, Helsinki, Finland; b Department of Applied Informatics, University of Macedonia, Thessaloniki, Greece; c Department of Physiology, School of Medicine, University of Patras, Patras, Greece; d School of Biomedical Engineering and Imaging Sciences, King’s College London, London, UK; e Department of Neurology & Stroke, Eberhard-Karls University, Tuebingen, Germany; f Hertie Institute for Clinical Brain Research, Eberhard-Karls University, Tuebingen, Germany

**Keywords:** Cerebral hemorrhage, oral anticoagulants, warfarin, direct factor Xa inhibitors, antithrombins

## Abstract

**Background:**

We investigated population-wide changes in prescriptions of oral anticoagulants (OAC) (vitamin K antagonists [VKA]) compared to direct oral anticoagulants (DOAC) and whether these translated into a decline in incidences of OAC-related intracerebral hemorrhages (ICH).

**Methods:**

This observational study analyzed OAC use in Southern Finland during 2005–2009 (pre-DOAC era) and 2015–2019 (transition-to-DOAC era) based on national registries. From medical records, we identified all non-traumatic ICH patients admitted to our tertiary hospital, the only one admitting neurological emergencies within the region. We compared the impact of DOACs versus VKAs on annual ICH incidence using an adjusted generalized additive model (GAM).

**Results:**

The total population increased from 1.4 to 1.7 million between 2005 and 2019. The use of OACs increased from 1.65% of the population in 2005–2009 (100% VKA) to 2.97% in 2015–2019 (64% VKA, 36% DOAC), resulting in 368,055 person-years of OAC use. In 2019, DOAC use exceeded VKA use. The annual incidence of ICH was 12/100,000 person-years in both periods. During 2005–2009, 115 (13%) of 917 ICHs were OAC-related (100% VKA) compared to 178 (18%) of 1002 ICHs in 2015–2019 (73% VKA, 27% DOAC). In the GAM, the rate of ICH among patients on DOACs was 53% lower than among patients on VKA (*p* < 0.001).

**Conclusions:**

ICH incidence remained stable despite near-doubled OAC use. The lower ICH rates with DOACs compared to VKAs support their safety advantage.

## Introduction

Direct oral anticoagulants (DOACs) are at least as effective as vitamin K antagonists (VKAs) in preventing ischemic stroke in patients with atrial fibrillation (AF) with lower rates of symptomatic intracerebral hemorrhage (ICH) reported in randomized controlled trials (RCTs). A meta-analysis of four RCTs (ROCKET AF [1], RE-LY [2], ARISTOTLE [3], ENGAGE AF-TIMI 48^4^) confirmed the favorable risk-benefit profile of DOACs with significant reductions in stroke, ICH, and mortality, and a similar incidence of extracranial major bleedings compared to warfarin [4]. Additionally, the benefit of DOACs in reducing thromboembolic complications with reduced or similar bleeding risk in treatment and prevention of venous thromboembolism, including prevention of thromboembolism after knee- and hip replacement surgery, has been demonstrated [5,6]. DOACs might be safer and more effective than VKAs due to their more selective mechanism of action and predictable pharmacology [7]. Indications for DOACs today also cover chronic coronary artery disease and peripheral artery disease [8]. Due to a more favorable risk-benefit profile, DOACs have become the preferred option for OACs, and their use has exceeded VKA’s [9]. However, further data on their safety and efficacy in routine clinical practice are still needed, especially regarding ICH.

In this study, we compared population-wide changes in OAC prescription patterns between two cohorts, 2005–2009 and 2015–2019, and examined how OAC use associates with admissions of patients with OAC-related ICH (OAC-ICH) in Southern Finland. Combining data from exhaustive national health registries and hospital data, we had a unique opportunity to monitor changes in OAC prescription patterns and ICH events over the study period.

## Patients and methods

We included all consecutive ICH patients admitted during 2005–2009 and 2015–2019 to the Department of Neurology, HUS Helsinki University Hospital, due to an index event of non-traumatic ICH and with a permanent residency in Uusimaa province, Southern Finland. The study site is the only hospital in the province with a 24/7 neurology emergency room (ER) and a stroke unit. Patients with acute stroke symptoms (i.e. stroke code patients) are admitted directly to the hospital unless they have severe pre-stroke dependency and frailty. The admission criteria remained unchanged during the study period.

The patients were systematically screened from the hospital patient records covering all hospital admissions. The diagnosis of non-traumatic ICH was confirmed from the medical records and brain scans. We excluded patients with primary subdural or epidural hematoma, traumatic ICH, hemorrhages related to a tumor, primary subarachnoid hemorrhages, hemorrhages due to reversible cerebral vasoconstriction syndrome, and hemorrhagic transformation of cerebral infarction. Hence, we included ICHs related to hypertensive or amyloid angiopathy, an underlying structural vascular pathology such as arteriovenous malformation or cavernoma, coagulopathy, or antithrombotic medication.

We attributed ICH to VKA treatment if the International Normalized Ratio (INR) was <2.0 on admission [10]. Patients with INR <2.0 were considered non-OAC-ICH. We excluded patients who, based on medical records, were on VKA but for whom INR was not available. ICH was considered DOAC-related (DOAC-ICH) if use at the time of the ICH was reported in the medical records, i.e. patient or caregiver confirmed medication use or anti-Xa assay for apixaban and rivaroxaban, or functional concentration of dabigatran confirmed use, or data on recent DOAC dispensation was reported in the electronic medication registry. Thus, DOAC use relied on prescription records and clinical documentation without routine laboratory confirmation.

Data on all OACs dispatched from pharmacies in the province were retrieved from the Social Insurance Institution of Finland’s registries, which cover all medical reimbursements in Finland. These data were reported annually per sex and age group for each DOAC (apixaban, dabigatran, edoxaban, and rivaroxaban) and warfarin, the only generally available VKA in Finland. The population of the province for 2005–2009 and 2015–2019 was obtained from Statistics Finland.

The Institutional Review Board of Helsinki University Hospital, Brain Center, approved this study and its analysis plan before initiating the study (HUS/216/2023). As an observational registry study with no study-related patient contact, based on the Act on the Secondary Use of Health and Social Data (552/2019), no consent from the participants was required. Further, separate approval from the ethical review board was not required because the research is not medical research as defined in the Medical Research Act. The study adheres to the Declaration of Helsinki.

### Statistical analyses

Categorical variables are presented as counts and valid percentages (%), whereas continuous variables are represented as median and interquartile range (IQR) values. Differences between the groups were evaluated using the chi-squared test or the Mann–Whitney U test in the case of two groups, or the one-way ANOVA in the case of three groups.

We compared the incidence of ICH among patients on VKA and DOAC using incidence rate ratios (IRRs) and a Generalized Additive Model (GAM) with a negative binomial distribution. As the primary analysis, we compared VKA-ICH and DOAC-ICH, and as an exploratory analysis, we also compared individual DOACs. The GAM was chosen considering its flexibility to capture non-linear associations between predictor variables and outcomes. This is particularly relevant in our dataset, where the incidence of ICH can vary significantly across age groups and over time. In the GAM, the number of annual events (i.e. ICH) per sex and age group was the dependent variable, and the type of OAC was the independent variable of interest. A smoothing function was used, including coefficients for gender and age group variables. The smoothing function was modeled randomly, allowing for group deviations. The total number of patients on OACs (number of receivers) was included as an offset variable (logarithmic scale) with a fixed coefficient of 1. An additional offset variable was introduced (the ratio of patients taking no OACs and having the event). Only patients receiving OACs were analyzed using GAM analysis. The percentage change comparing DOAC to VKA was calculated using the formula:

$$\text{Percentage Change}=(e^{\text{Coefficient}}-1)*100$$

Statistical analysis was conducted with R-Studio 6.1.0 (Posit Software, PBC, Boston). A two-sided probability (*p*) value < 0.05 was considered significant.

## Results

### Description of the two patient cohorts

**Figure 1.**
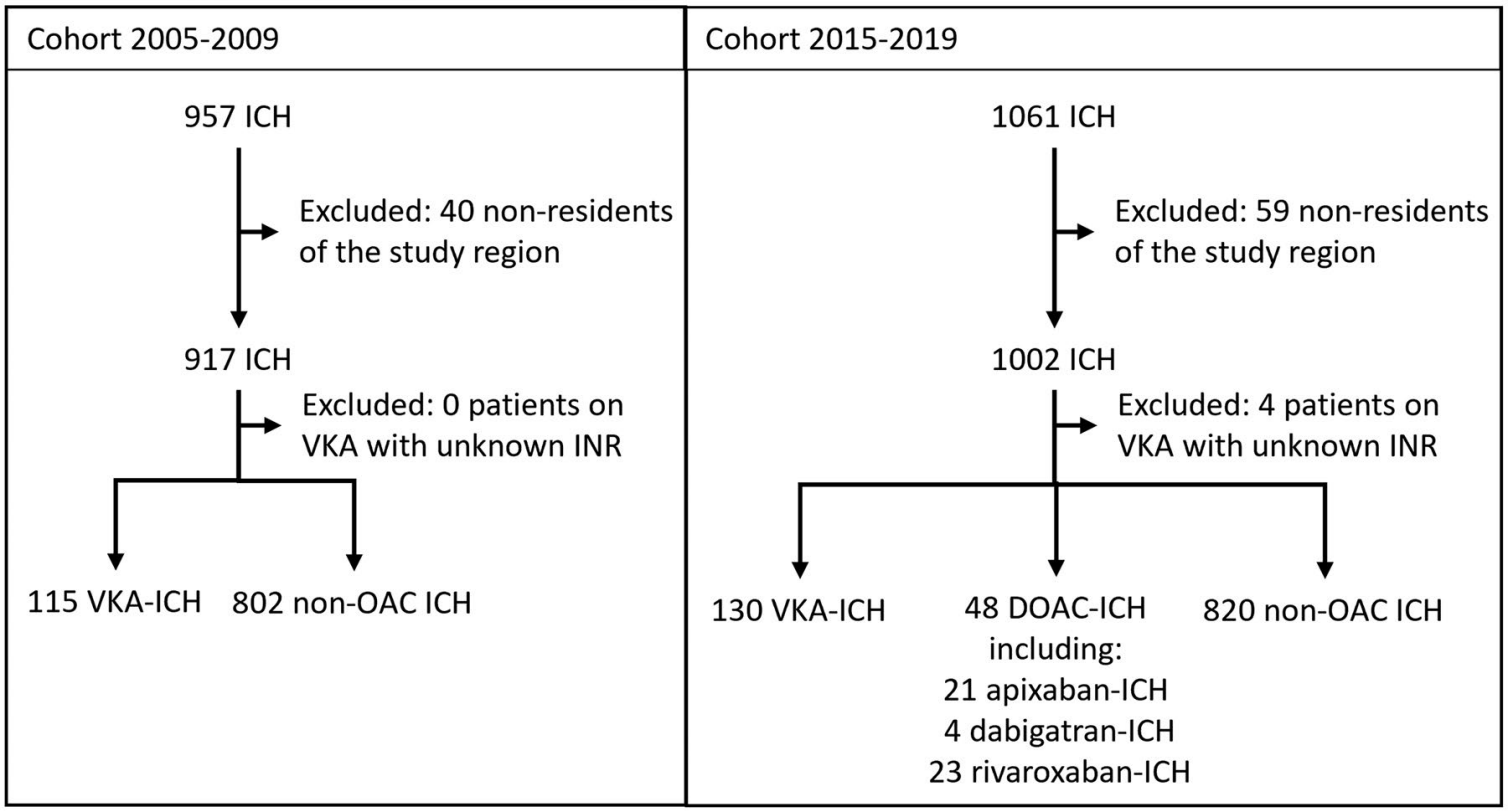
Flowchart of patient inclusion for ICH patient cohorts from 2005–2009 and 2015–2019. Among the eligible patients, non-residents of the study region and those with unknown INR values were excluded. ICH: intracerebral hemorrhage; OAC: oral anticoagulant; VKA: vitamin K antagonist; DOAC: direct oral anticoagulant.

The 2005–2009 cohort (pre-DOAC era) consisted of 917 ICH patients after excluding 40 non-residents of the hospital’s catchment area. In this cohort, the ICHs were classified as VKA-related ICH (VKA-ICH) and non-VKA-related (non-VKA-ICH). VKA was used by 115 (12.5%) patients (Figure 1). Compared to non-VKA-ICH, those with VKA-ICH were older (median 76.5, IQR 71.0–82.0 vs. 66.5, 57.0–77.0, *p* < 0.001), more often males (67.8% vs. 55.6%, *p* = 0.013). Additional basic characteristics of the patients are depicted in Table 1 and Supplementary Table 1.

**Table 1.** Baseline characteristics of ICH patients during two study periods (2005–2009 and 2015–2019).

	No OAC^a^	VKA	DOAC	*p* value	Missing
**2005–2009**					
Total *n*	802	115	0		
Age, years	66.5 (57.0–77.0)	76.5 (71.0–82.0)	NA	**<0.001**	
Female	356 (44.4)	37 (32.2)	NA	**0.013**	
**2015–2019**					
Total *n*	820	130	48		4^c^
Age, years	70.0 (58.0–78.0)	78.5 (72.0–84.0)	77.0 (71.5–81.0)	**<0.001**	
Female	392 (47.8)	52 (40.0)	27 (56.3)	0.111	

Data are expressed as n (%) or median (IQR). Significant *p* values (<0.05) are in bold.

ICH: intracerebral hemorrhage; INR: International Normalized Ratio; IVH: intraventricular hemorrhage; OAC: oral anticoagulant; VKA: vitamin K antagonist; DOAC: direct oral anticoagulant; NA: not applicable.

^a^ Includes patients with VKA and INR < 2.0.

^b^ 14 no OAC and 1 VKA group.

^c^ unknown INR value.

The 2015–2019 cohort (transition-to-DOAC era) comprised 1002 patients after excluding 59 non-residents of the catchment area. DOAC-ICH accounted for 48 (4.8%) patients, including 21 (43.8% of DOAC-ICH) apixaban-related, 4 (8.3%) dabigatran-related, and 23 (48.0%) rivaroxaban-related ICHs. One patient who was prescribed DOAC was classified as non-OAC-ICH because medical records stated the patient was not using the medication. There were no patients with a DOAC prescription for whom we could not verify the use (or non-use) in the medical records. VKA-ICH accounted for 130 (13.0%) patients (Figure 1). Four (0.4%) patients reported to be on VKA did not have an INR available and were excluded. Patients with OAC-ICH (either on VKA or DOAC) were older (78.0, 72.0–84.0 and 77.0, 71.5–81.0, respectively) than non-OAC-ICH patients (70.0, 58.0–78.0, *p* < 0.001). Table 1 and Supplementary Table 2 show the patient characteristics for each subgroup, and Supplementary Table 3 displays dosages on individual DOACs.

The most common indication for VKA (80.0% of the VKA-ICH patients in 2005–2009 and 78.8% in 2015–2019) was AF, followed by pulmonary embolism and deep venous thrombosis (10.4% of the VKA-ICH patients in 2005–2009 and 8.0% in 2015–2019), and mechanical heart valve (3.6% of the VKA-ICH patients in 2005–2009 and 3.7% in 2015–2019). Other indications were rare (Supplementary Table 4). Among DOAC-ICH patients, the indication was virtually solely AF (97.9%), while only one (2.1%) patient had a pulmonary embolism or deep venous thrombosis as the indication.

### Annual incidence of ICH per 10,000 patients on OACs

The total population in our catchment area increased from 1.4 million to 1.7 million from 2005 to 2019. In the province, the annual incidence of ICH (irrespective of the OAC status) was 12 per 100,000 inhabitants in both cohorts (2005–2009 and 2015–2019).

**Figure 2.**
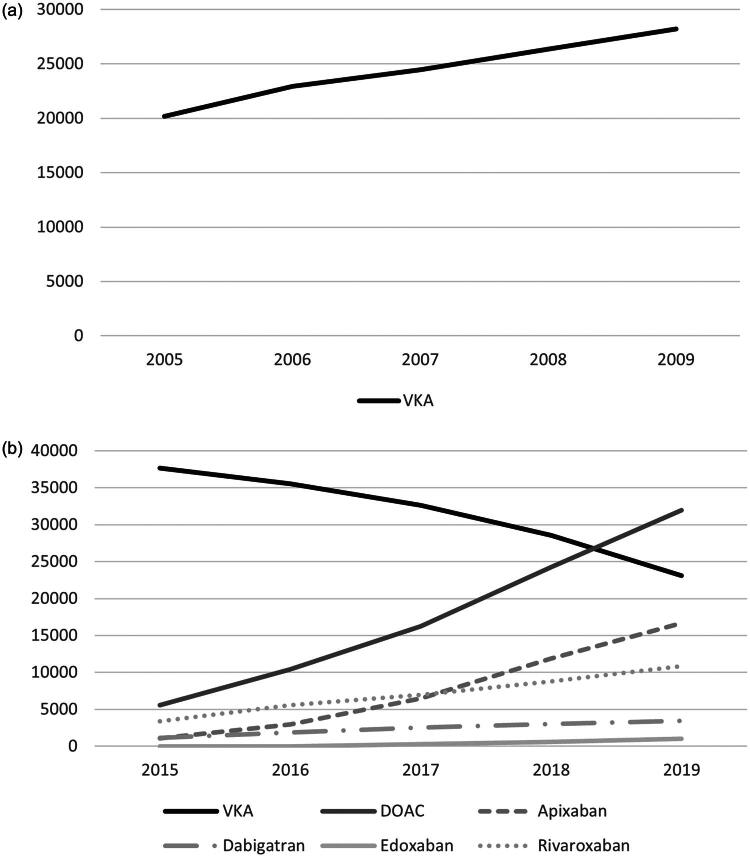
Total number of patients on OACs in the study region during (a) 2005–2009 (100% VKA) and (b) 2015–2019 (64% VKA, 36% DOAC). The Y-axis shows the number of patients, and the X-axis shows the year. OAC: oral anticoagulant; VKA: vitamin K antagonist; DOAC: direct oral anticoagulant.

Between 2005 and 2009, the yearly number of patients on OACs increased from 20,187 to 28,193 (100% VKA). From 2015 to 2019, the number increased from 43,257 to 55,106 (64% VKA, 36% DOAC), and in 2019 patients on DOACs exceeded patients on VKA. In total, we observed 368,055 person-years of OAC use. Figure 2 depicts annual changes in OAC use in our province between 2005 and 2009 and 2015–2019.

The annual incidence of ICH per 10,000 patients on VKA was 9.4 during 2005–2009 and 8.3 during 2015–2019 (IRR 0.87, 95% CI 0.68–1.12, *p* = 0.304). In the 2015–2019 cohort, there were 5.4 DOAC-related ICHs per 10,000 patients on DOACs per year, and compared to patients on VKA during the same period, the incidence rate was 34.2% lower among patients on DOACs (IRR 0.66, 95% CI 0.47–0.91, *p* = 0.011). Tables 2–4 depict the annual population of our province, the number of ICH patients, the number of patients on OACs, and the number of OAC-ICH patients. These tables also show the overall incidence of any ICH (irrespective of the OAC status) and the incidence of ICH among the patients on OACs in our population.

**Table 2.** Population size and the number of all ICH patients, patients on OACs, and VKA-related ICH in 2005–2009.

Year	Population, *n*	ICH, *n* (*n*/100,000)	Patients on VKA, *n* (%)	VKA-ICH, *n* (*n*/10,000 users)
2005	1,448,026	175 (12)	20,187 (1.39)	20 (9.9)
2006	1,463,385	179 (12)	22,920 (1.57)	27 (11.8)
2007	1,479,671	190 (13)	24,463 (1.65)	29 (11.9)
2008	1,497,476	199 (13)	26,363 (1.76)	20 (7.6)
2009	1,513,517	174 (11)	28,193 (1.86)	19 (6.7)

ICH: intracerebral hemorrhage; OAC: oral anticoagulant; VKA: vitamin K antagonist.

**Table 3.** Population size and the number of all ICH patients, patients on OACs, and OAC-related ICH in 2015–2019.

Year	Population, *n*	ICH, *n* (*n*/100,000)	Patients on DOAC, *n* (%)	Patients on VKA, *n* (%)	DOAC-ICH, *n* (*n*/10,000 users)	VKA-ICH, *n* (*n*/10,000 users)
2015	1,616,321	170 (11)	5568 (0.34)	37,689 (2.33)	3 (5.4)	26 (6.9)
2016	1,634,319	195 (12)	10,408 (0.64)	35,508 (2.17)	6 (5.8)	30 (8.4)
2017	1,651,715	207 (13)	16,226 (0.98)	32,629 (1.98)	11 (6.8)	29 (8.9)
2018	1,667,203	233 (14)	24,250 (1.45)	28,545 (1.71)	15 (6.2)	31 (10.9)
2019	1,685,983	193 (11)	31,985 (1.90)	23,121 (1.37)	13 (4.1)	14 (6.1)

Data are presented as *n* (*n*/100,000 inhabitants), *n* (% of the entire population), or *n* (*n*/10,000 users). ICH: intracerebral hemorrhage; OAC: oral anticoagulant; DOAC: direct oral anticoagulant; VKA: vitamin K antagonist.

**Table 4.** The total number of patients on DOACs and direct OAC-related ICH in 2015–2019.

Year	Patients on Apixaban, *n* (%)	Patients on Dabigatran, *n* (%)	Patients on Edoxaban, *n* (%)	Patients on Rivaroxaban, *n* (%)	Apixaban-ICH, *n* (*n*/10,000 users)	Dabigatran-ICH, *n* (*n*/10,000 users)	Edoxaban-ICH, *n* (*n*/10,000 users)	Rivaroxaban-ICH, *n* (*n*/10,000 users)
2015	1066 (0.07)	1113 (0.07)	0	3389 (0.21)	0	1 (9.0)	0	2 (5.9)
2016	2941 (0.18)	1887 (0.12)	4	5576 (0.34)	2 (6.8)	0	0	4 (7.2)
2017	6649 (0.39)	2552 (0.15)	264 (0.02)	6941 (0.42)	10 (15.5)	0	0	1 (1.4)
2018	11,882 (0.71)	3019 (0.18)	576 (0.03)	8773 (0.53)	4 (3.4)	3 (9.9)	0	8 (9.1)
2019	16,688 (0.99)	3456 (0.20)	1021 (0.06)	10,820 (0.64)	5 (3.0)	0 (0)	0	8 (7.4)

Data are presented as *n* (% of the entire population) or *n* (*n*/10,000 DOAC users). ICH: intracerebral hemorrhage; DOAC: direct oral anticoagulant; OAC: oral anticoagulant.

In the multivariable GAM adjusted for age, sex, and year of treatment, we found that DOACs decreased the rate of ICH by 52.7% (95% CI of percentage change −63.0% to −39.6%) compared with VKA (Table 5).

**Table 5.** Summary table of generalized additive model results.

Family: negative binominal	Coefficient	Std. Error	*p* value	Percentage change	95% CI percentage change
DOAC	−0.749	0.125	**<0.001**	−52.7	−63.0 to −39.6

DOAC: direct oral anticoagulant; VKA: vitamin K antagonist. Significant *p* values (<0.05) are in bold.

### Sensitivity and exploratory analyses

In the exploratory analyses regarding individual DOACs, the annual incidences per 10,000 patients on apixaban, dabigatran and rivaroxaban were 5.4 (IRR 0.65, 95% CI 0.41–1.03, *p* = 0.060 compared to VKA), 3.3 (IRR 0.40, 95% CI 0.14–1.09, *p* = 0.048 compared to VKA), and 6.5 (IRR 0.78, 95% CI 0.50–1.22, *p* = 0.283 compared to VKA), respectively. The number of patients on edoxaban was very low, and none of the edoxaban-treated patients suffered an ICH.

In the multivariable GAM, when each DOAC was analyzed separately (Supplementary Table 5), we observed that each DOAC decreased the rate of ICH compared to VKA, with the following percentage change (95% CI of percentage change): apixaban −54.4% (−67.0% to −37.0%), dabigatran −78.3% (−91.9% to −41.7%) and rivaroxaban −38.6% (−56.3% to −13.8%). Because there was no edoxaban-related ICH, it was not analyzed separately. Given the low number of events per individual DOAC and thus lower statistical power, it is important to interpret these results as exploratory. Especially, the dabigatran analysis was based on only 4 events; the CI crosses the null (0.14–1.09); thus, the results remain insufficient for clinical inference.

As a sensitivity analysis, we assigned all patients with reported VKA use, irrespective of their INR values, to the VKA-ICH category. This resulted in 130 (14.2%) VKA-ICH in 2005–2009 and 150 (15.0%) VKA-ICH in 2015–2019. All DOAC categories were associated with a lower likelihood of ICH compared with VKA (reference category) (data not shown).

## Discussion

In the present study, we investigated ICH incidence and the prescription patterns of different OACs in two very distinct cohorts. The first cohort represents the time before the era of DOACs. The second cohort covers a very important epoch, namely, after the update of the international guidelines in 2014 [11,12], the transition from VKA to DOACs, the changes in their reimbursement policy, and the fact that DOAC prescriptions exceeded those of VKA by 2019. This study setting provided a unique opportunity to assess the transition of OAC use and the incidence of ICH in the largest province in Finland, which captures 1/3 of the Finnish population – an unselected population cohort of nearly 1.7 million residents. Since Helsinki University Hospital is the only neurological ER in the province and the admission criteria remained the same throughout our study, it enabled comparison of admissions throughout the study periods. The Finnish national registries have accurate population counts and detailed information on the dispensed drugs. Thus, our results provide important complementary information to previous studies on the impact of OAC use on ICH incidence. In our study population, the incidence of ICH remained stable over time, and the rate of ICH was approximately one-half lower among patients on DOACs than it was among patients on VKA.

In line with other reports, the use of OACs (as a percentage of the population) nearly doubled between 2005 and 2009 and 2015 and 2019 [13–15]. This is likely due to several factors, including an aging population and more active preventive treatments in AF patients. Additionally, DOACs gained an indication for thrombosis prophylaxis after hip and knee replacement surgery, which warfarin does not have. Despite the increase in the number of patients on OACs, the overall incidence of ICH remained stable in our cohort. In addition to the shift from VKA to DOAC, the stability of the ICH incidence in our cohorts probably also reflects the overall trend of decreasing ICH incidence, which is most likely attributable to several factors, such as developed healthcare systems and early intervention measures [16].

Earlier studies have reported varying incidence of ICH during the past decades—in some cohorts the incidence remained stable, and in others decreased [12–14]. These variations can be explained by different populations, time periods, age groups, cohort sizes, and analysis methods. Nonetheless, studies focused particularly on OAC-ICH incidence are scarce.

Previous reports on the safety and the ICH incidence of OAC are mainly from cohorts of patients with AF. A meta-analysis of more than 82,000 AF patients comparing the risk of ICH associated with VKA or DOAC showed that DOACs reduced the risk of ICH by almost half compared to VKA [17]. In a study of more than 434,000 patients on OACs with non-valvular AF, apixaban, dabigatran, and rivaroxaban were associated with lower rates of intracranial hemorrhage, including also subarachnoid hemorrhage and traumatic hemorrhage [18]. A study of more than 420,000 AF patients from Europe and Canada showed that DOACs compared to VKAs did not increase the risk of bleeding, and for intracranial hemorrhages, all individual DOACs reduced the risk for bleeding [19]. A Danish case-control study of almost 17,000 ICH patients investigated the association between antithrombotic use and ICH and found the strongest association with VKA (cases: 12.0%, controls: 5.0%), and much lower with DOACs (cases: 3.0%, controls 1.8%) [14].

In a general population, a Dutch study comparing two 3-year periods (2007–2009 vs 2017–2019) showed a decrease in total number of ICH and OAC-ICH incidence, despite the aging population and increase in OAC use [13]. A Spanish study found a 3,7-fold increase in annual OAC-ICH incidence from 2008 to 2018, which was attributed at least partially by increasing age [20]. Our results are in line with these studies, but importantly, our cohort also provided information on actual OAC-use in the population enabling comparison of OAC-use related ICH incidence. Thanks to the detailed clinical data, we could monitor the efficacy of VKA use with INR values and exclude patients with no or weak treatment effects while capturing the patients with actual anticoagulation effects.

For four individual DOACs (apixaban, dabigatran, rivaroxaban, edoxaban), a multinational population-based study covering 527,226 patients on DOACs (AF patients) found that apixaban was associated with a lower risk for gastrointestinal bleeding, while no substantial differences were observed in other outcomes (including ischemic stroke, ICH and all-cause mortality) [21]. A network meta-analysis of 82,404 AF patients from RCTs found, however, that DOACs nearly halved the risk of ICH compared to VKA, but rivaroxaban was relatively unsafe compared to dabigatran; dabigatran 110 mg was associated with 53% lower relative risk for ICH than rivaroxaban 20 mg [17]. Also, dabigatran was associated with the lowest risk for ICH in our cohort. It is possible that patients with previous morbidity, including renal failure and previous gastrointestinal issues, were more likely to be on VKA or other DOACs than dabigatran. Thus, observed differences between DOACs and VKA may reflect prescribing patterns rather than actual pharmacological differences. Furthermore, our sample sizes remain limited for drawing definitive conclusions when comparing different DOACs, and effect estimates for individual DOACs should be considered exploratory. However, our results from over 88,000 patients on DOACs in the general population, irrespective of indication for DOAC use, are consistent with previous results. Larger sample sizes will be needed to more effectively demonstrate potential differences in the safety profiles between different DOACs.

A retrospective study on prescription patterns and outcomes of AF patients and their OAC prescriptions found that 45% of new OAC prescriptions were DOACs. However, patients with higher CHA_2_DS_2_VASc scores were more likely to receive warfarin [22]. Interestingly, a prospective RCT of 1323 elderly (>75 years) and frail AF patients showed that switching an INR-guided VKA therapy to DOAC led to more bleeding complications than continued use of VKA [23]. Indeed, in routine clinical practice, the prescription patterns may also be affected by comorbidities, such as valvular diseases, which may demand VKA treatment with more intense anticoagulation. Among our DOAC-ICH patients, the indication for DOAC use was almost solely AF, whereas VKA-ICH patients had a wider spectrum of indications for their VKA use. Thus, residual confounding by indication may bias our estimates toward a greater apparent DOAC benefit.

DOACs may be safer and more effective than VKAs due to their more selective mechanism of action and predictable pharmacological profile [7]. Clinically, large randomized trials and meta-analyses demonstrated that DOACs are at least non-inferior to warfarin for stroke prevention in atrial fibrillation, while conferring a significantly lower risk of intracranial hemorrhage—a significant determinant of anticoagulant safety [1–3,7,24]. Our results from the general population add to the growing body of evidence that DOACs should be preferred over VKAs in clinical settings where DOACs have an established indication. Current European guidelines recommend DOACs over VKA in the prevention of ischemic stroke and thromboembolism in patients with AF [12,25,26]. In addition to treatment guidelines, reimbursement status and criteria seem to be important factors in DOAC use. In Finland, DOAC use has increased slightly more slowly than in many other countries, maybe due to the reimbursement policy [15]. Residual risk of ICH and other severe bleeding events persists even with DOAC use and future research should focus on identifying and modifying bleeding risk factors among patients on DOACs. Tools such as the DOAC Score have highlighted modifiable factors, including hypertension, diabetes, renal impairment, and concomitant use of antiplatelet or NSAID medications [27]. Additionally, dose optimization strategies—such as reduced-dose regimens for secondary prevention—have shown promise in lowering bleeding rates without compromising efficacy [28].

### Limitations

Our study has limitations that should be considered. First, we could only include patients admitted to Helsinki University Hospital. It is possible that a small number of residents in our province were hospitalized for ICH at other hospitals (e.g. while traveling). However, most likely, we caught such patients in the rehabilitation phase. Also, patients with significant frailty and dependence on activities of daily living were admitted to local hospitals. However, the transfer criteria for emergency medical services remained the same throughout the study period, enabling a reliable comparison of hospital admissions during the two study periods. Second, due to our study’s retrospective design, compliance with DOAC treatment was not systematically verified by a laboratory test (as compared to INR for VKA), but was instead verified from medical and prescription records. This introduces a potential misclassification bias that favors DOACs if patients are erroneously classified as DOAC users. However, it is important to note that routine laboratory monitoring is not performed for DOACs, unlike for VKA. Since we included only local residents, we were able to reliably track all the OAC prescriptions in the prescription records, further improving the reliability of DOAC use. Third, the reimbursement data could not exclusively disclose all indications of OAC use, and for VKA, we did not have indications available. Thus, we lack precise information on DOAC dose and duration of use. Further, there might be an overlap in the number of users among individuals who changed OAC during the year. Unlike VKAs, DOACs can be used for short periods and/or at reduced doses (postoperative DVT prophylaxis or prevention of atherothrombotic events [rivaroxaban only]). However, based on the reimbursement data, most patients on DOACs had AF as the indication for their use, indicating long-term use (Supplementary Table 4). In our patient cohort, we were unable to evaluate dose appropriateness, which may influence bleeding risk, because of missing data for some patients (Supplementary Table 3). Also worth noting is that, in addition to OAC use, many other factors likely contribute to ICH incidence. For example, changes in blood pressure management practices over the study periods may affect the overall incidence of ICH in the population [29]. In our study, we were unable to address such population-level factors as well as overall estimation of the bleeding risk using, for example, CHA_2_DS_2_-VASc/HAS-BLED scores. Also, it is important to acknowledge the limited sample size regarding ICH events and individual DOACs, especially dabigatran. Despite contractual independence, minimal dabigatran events (*n* = 4) warrant heightened skepticism regarding 78% risk reduction claim. Finally, because the two cohorts were separated in time and exposed to different background conditions, our design cannot establish causality. The results show an association rather than a causal effect.

## Conclusions

DOAC transition prevented the expected rise in population-level ICH despite doubled anticoagulant exposure—representing public health success. DOAC-related ICH occurred at roughly half the rate of VKA-related cases. Nevertheless, the stable incidence of OAC-associated ICH underscores the need for targeted strategies to reduce residual bleeding risk among all patients on OACs, such as the DOAC score and blood pressure optimization [27]. Our findings support the superior safety profile of DOACs compared with VKAs. With less restrictive compensation policies for DOACs, switching from VKAs to DOACs when clinically appropriate should eventually result in fewer OAC patients experiencing ICH.

## Supplementary Material

Oral anticoagulant use and ICH incidence supplementary material.docx

## Data Availability

The data supporting this study’s findings are available from the corresponding author upon reasonable request.
