# Supplementary material for: Changes in the use of vitamin K antagonists and direct oral anticoagulants and impact on the incidence of oral anticoagulation-related intracerebral hemorrhage: population-wide prescription patterns in two 5-year cohorts
Source: Ann Med. 2026 Apr 6;58(1):2652115. doi: 10.1080/07853890.2026.2652115 (PMC13055024; doi:10.1080/07853890.2026.2652115)
Supplement: Oral anticoagulant use and ICH incidence supplementary material.docx [file IANN_A_2652115_SM5396.docx]

**Supplementary material to Changes in the use of vitamin K antagonists and direct oral anticoagulants and impact on the incidence of oral anticoagulation-related intracerebral hemorrhage: population-wide prescription patterns in two 5-year cohorts**

**Supplementary Table 1.** Characteristics of the ICH patients in the 2005-2009 cohort

|  | No OAC | VKA | P-value | Missing cases (No OAC / VKA) |
| --- | --- | --- | --- | --- |
| **2005 (n=175)** | n=155 | n=20 |  |  |
| Age, y (IQR) | 67.0 (56.0-77.5) | 78.5 (74.0-83.5) | **<0.001** |  |
| Female (%) | 68 (43.9) | 5 (25.0) | 0.148 |  |
| **2006 (n=179)** | n=152 | n=27 |  |  |
| Age, y (IQR) | 67.5 (58.5-78.0) | 76.0 (67.5-81.5) | **0.009** |  |
| Female (%) | 54 (35.5) | 10 (37.0) | 1.000 |  |
| **2007 (n=190)** | n=161 | n=29 |  |  |
| Age, y (IQR) | 69.0 (57.0-78.0) | 78.0 (72.0-82.0) | **<0.001** |  |
| Female (%) | 78 (48.4) | 13 (44.8) | 0.841 |  |
| **2008 (n=199)** | n=179 | n=20 |  |  |
| Age, y (IQR) | 64.0 (57.0-77.0) | 74.5 (65.5-79.5) | 0.0097 |  |
| Female (%) | 83 (46.46) | 6 (30.0) | 0.163 |  |
| **2009 (n=174)** | n=155 | n=19 |  |  |
| Age, y (IQR) | 68.0 (59.0-75.0) | 76.0 (68.0-81.0) | **0.007** |  |
| Female (%) | 73 (47.1) | 3 (15.8) | **0.013** |  |

Data are expressed as n (%) or median (IQR). Significant P-values (<0.05) are in bold.

ICH = intracerebral hemorrhage; INR = International Normalized Ratio; IVH = intraventricular hemorrhage; VKA = vitamin K antagonist

**Supplementary Table 2.** Characteristics of the ICH patients in the 2015-2019 cohort.

|  | No OAC | VKA | DOAC | P-value |
| --- | --- | --- | --- | --- |
| **2015 (n=170)** | n=141 | n=26 | n=3 |  |
| Age, y (IQR) | 69.0 (58.0-79.0) | 79.5 (77.0-82.0) | 76.0 (73.0-81.5) | **<0.001** |
| Female (%) | 69 (48.9) | 17 (65.4) | 2 (66.7) | 0.266 |
| **2016 (n=194)** | n=158 | n=30 | n=6 |  |
| Age, y (IQR) | 68.0 (57.0-75.0) | 76.0 (69.0-82.0) | 79.0 (75.0-79.0) | **0.001** |
| Female (%) | 70 (44.3) | 8 (26.7) | 4 (66.7) | **0.094** |
| **2017 (n=206)*** | n=166 | n=29 | n=11 |  |
| Age, y (IQR) | 69.5 (57.0-78.0) | 78.0 (74.0-84.0) | 78.0 (76.0-81.5) | **<0.001** |
| Female (%) | 80 (48.2) | 15 (51.7) | 6 (54.5) | 0.876 |
| **2018 (n=235)*** | n=189 | n=31 | n=15 |  |
| Age, y (IQR) | 71.0 (59.0-78.0) | 80.0 (69.5-86.0) | 72.0 (70.5-77.0) | **0.002** |
| Female (%) | 91 (48.1) | 9 (29.0) | 10 (66.7) | **0.040** |
| **2019 (n=193)** | n=166 | n=14 | n=13 |  |
| Age, y (IQR) | 71.5 (60.0-78.0) | 78.5 (72.0-84.0) | 78.0 (69.0-82.0) | **0.004** |
| Female (%) | 82 (49.4) | 3 (21.4) | 5 (38.5) | 0.105 |

Data are expressed as n (%) or median (IQR). Significant P-values (<0.05) are in bold.

*four patients with on warfarin but with missing INR excluded (two in 2017 and two in 2018)

ICH = intracerebral hemorrhage; INR = International Normalized Ratio; IVH = intraventricular hemorrhage; DOAC = direct oral anticoagulant, OAC = oral anticoagulant, VKA = vitamin K antagonist

**Supplementary Table 3.** Dosages of the DOACs in the 2015-2019 cohort.

| DOAC and dosage | N (% of users) |
| --- | --- |
| Dabigatran 110 mg b.i.d. | 1 (2.1) |
| Dabigatran 150 mg b.i.d. | 3 (6.3) |
| Apixaban 2.5 mg b.i.d | 3 (6.3) |
| Apixaban 5 mg b.i.d | 17 (35.4) |
| Apixaban UNK | 1 (2.1) |
| Rivaroxaban 20 mg q.d. | 18 (37.5) |
| Rivaroxaban 15 mg q.d. | 1 (2.1) |
| Rivaroxaban UNKf | 4 (8.3) |

DOAC = direct oral anticoagulant; b.i.d = twice a day; q.d. = once a day; UNK = unknown.

**Supplementary Table 4.** Indication for anticoagulation among the ICH patients.

|  | Atrial fibrillation | Pulmonary embolism/deep venous thrombosis | Mechanical heart valve | Other | Thrombosis prophylaxis | Indication unknown |
| --- | --- | --- | --- | --- | --- | --- |
| **2005-2009, n=115** |  |  |  |  |  |  |
| VKA, n=115 | 92 (80.0) | 12 (10.4) | 4 (3.6) | 4 (3.6)^1^ | 0 | 3(2.6) |
| **2015-2019, n=186** |  |  |  |  |  |  |
| VKA, n=130 | 107 (78.8) | 8 (8.0) | 5 (3.7) | 9 (7.3)^2^ | 0 | 1 |
| Apixaban, n=21 | 21 (100) | 0 | 0 | 0 | 0 | 0 |
| Dabigatran, n=4 | 4 (100) | 0 | 0 | 0 | 0 | 0 |
| Edoxaban, n=0 | N/A | N/A | N/A | N/A | N/A | N/A |
| Rivaroxaban, n=23 | 22 (95.6) | 1 (4.3) | 0 | 0 | 0 | 0 |

Data are expressed as n (%). N/A = not applicable; VKA = vitamin K antagonist

^1^aortic stenosis, activated protein C resistance, essential thrombocytosis, Fallot’s tetralogy

^2^membranous glomerulonephritis, phospholipid antibodies, nephrotic syndrome, left atrial appendage thrombus, dilated cardiomyopathy, patent foramen ovale, antiphospholipid antibodies and Budd-Chiari syndrome, bioprosthetic heart valve, ischemic stroke, cerebral venous thrombosis

**Supplementary Table 5.** Summary table of generalized additive model results for each DOAC.

| Family: negative binominal | Coefficient | Std. Error | P-value | Percentage change | 95 % CI percentage change |
| --- | --- | --- | --- | --- | --- |
| Apixaban | -0.786 | 0.165 | **<0.001** | -54.5 | -67.0 – -37.0 |
| Dabigatran | -1.526 | 0.503 | **0.002** | -78.3 | -91.9 – -41.7 |
| Rivaroxaban | -0.488 | 0.173 | **0.005** | -38.6 | -56.3 – -13.8 |

DOAC = direct oral anticoagulant; VKA = vitamin K antagonist. Significant P-values (<0.05) are in bold.
